# Supplementary figures and images for: Clostridium butyricum relieve the visceral hypersensitivity in mice induced by Citrobacter rodentium infection with chronic stress
Source: PeerJ. 2021 Jun 21;9:e11585. doi: 10.7717/peerj.11585 (PMC8223894; doi:10.7717/peerj.11585)

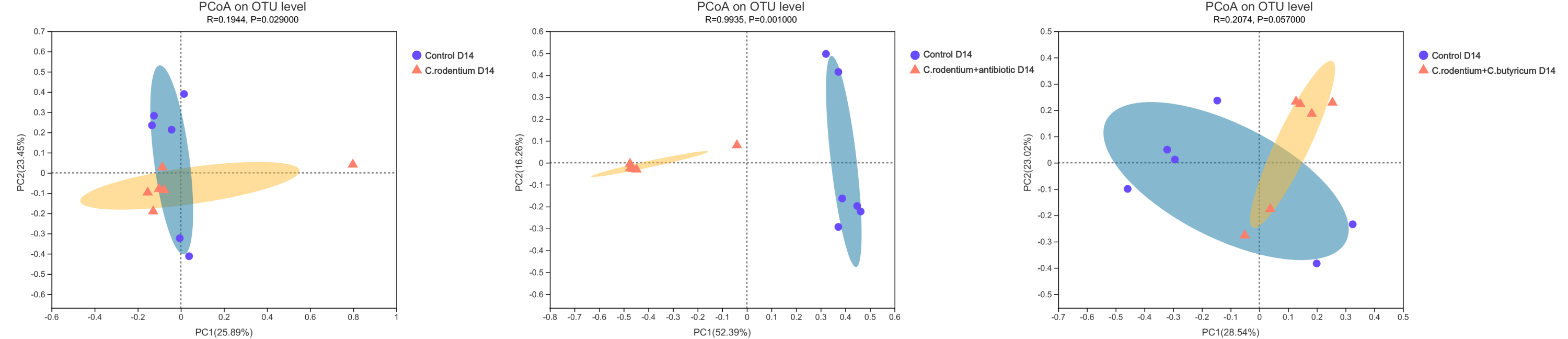

Supplement: Supplemental Information 1 — The structure of microbiota was similar between the C. rodentium infectious group and control group, which was also happened in the C. butyricum group. However, there were significant differences between the antibiotic-treatment group and control group (P < 0.05). [file peerj-09-11585-s001.png]

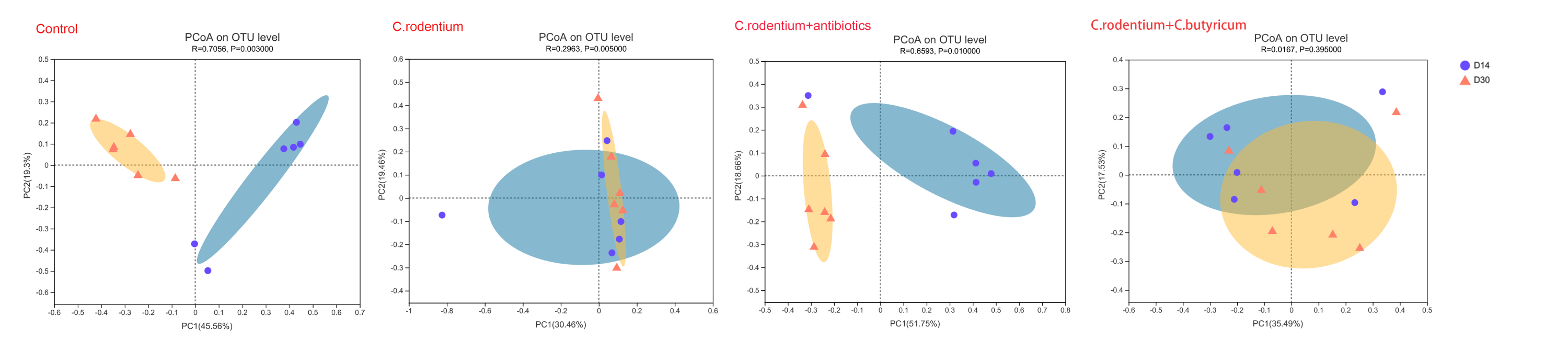

Supplement: Supplemental Information 2 — After the WAS at day 30, the difference between the antibiotic antibiotic-treatment group and the other three groups was further increased. However, the structure of the microbiota of the C. butyricum - treatment group did not change after WAS. [file peerj-09-11585-s002.png]
