# Supplementary material for: Clostridium butyricum relieve the visceral hypersensitivity in mice induced by Citrobacter rodentium infection with chronic stress
Source: PeerJ. 2021 Jun 21;9:e11585. doi: 10.7717/peerj.11585 (PMC8223894; doi:10.7717/peerj.11585)
Supplement: Supplemental Information 4 [file peerj-09-11585-s004.docx]

Table S1 The sample name and group information of 16S gene pyrosequencing

| Time | **Sample name** | | | | | |
| --- | --- | --- | --- | --- | --- | --- |
| Day 0 | **13D0** | **19D0** | **11D0** | **5D0** | **17D0** | **15D0** |
|  | **23D0** | **14D0** | **10D0** | **8D0** | **20D0** | **2D0** |
|  | **22D0** | **7D0** | **9D0** | **28D0** | **16D0** | **27D0** |
|  | **25D0** | **24D0** | **26D0** | **3D0** | **1D0** | **4D0** |
| Day 7 | **13D7** | **19D7** | **11D7** | **5D7** | **17D7** | **15D7** |
|  | **23D7** | **14D7** | **10D7** | **8D7** | **20D7** | **2D7** |
|  | **22D7** | **7D7** | **9D7** | **28D7** | **16D7** | **27D7** |
|  | **25D7** | **24D7** | **26D7** | **3D7** | **1D7** | **4D7** |
| Day 14 | **13D14** | **19D14** | **11D14** | **5D14** | **17D14** | **15D14** |
|  | **23D14** | **14D14** | **10D14** | **8D14** | **20D14** | **2D14** |
|  | **22D14** | **7D14** | **9D14** | **28D14** | **16D14** | **27D14** |
|  | **25D14** | **24D14** | **26D14** | **3D14** | **1D14** | **4D14** |
| Day 21 | **13D21** | **19D21** | **11D21** | **5D21** | **17D21** | **15D21** |
|  | **23D21** | **14D21** | **10D21** | **8D21** | **20D21** | **2D21** |
|  | **22D21** | **7D21** | **9D21** | **28D21** | **16D21** | **27D21** |
|  | **25D21** | **24D21** | **26D21** | **3D21** | **1D21** | **4D21** |

Red , *C.rodentium*+Antibiotic group; Blue, *C.rodentium* group; Green, *C.rodentium* +*C.butyricum* group; Black, Control group.
